# Minutes of the Third Annual Meeting of the Illinois State Medical Society, Held at Chicago, June, 1853

**Published:** 1853-06

**Authors:** 


### EDITORIAL.

*Minutes of the Third Annual Meeting of the Illinois State Medical Society, Held at Chicago, June,* 1853.

*June 7th,* 1853.

THE Illinois State Medical Society met in the Common Council Room, in the city of Chicago, at 10 A. M.

The President, Dr. ROUSE, being absent, Dr. THOS. HALL, the first Vice-President, took the Chair and called the Society to order.

The Committee of Arrangements, through their Chairman, Dr. MCARTHUR, welcomed to the city the members of the Society, after which, they reported the following list of Delegates from local Societies and Medical Institutions.

*Lisi of Delegates.*

| | |
|---|---|
| *Cook County Medical Society.* | Drs. J. V. Z. BLANEY, DE LASKIE MILLER, J. H. BIRD, ERIAL MCARTHUR, WM. E. CLARK. |
| *Stark County Medical Society,* | Drs. WM. CHAMBERLAIN, THOS. HALL. |
| *Rush Medical College,* | Drs. DANIEL BRAINARD, JOHN EVANS. |
| *Illinois General Hospital,* | Dr. N. S. DAVIS. |
| *U. S. Marine Hospital,* | Dr. W. B. HERRICK. |
| *Peoria City Medical Society,* | Dr. A. B. CHAMBERS. |
| *Winnebago Co. Med. Society,* | Drs. LUCIUS CLARK, WM. LYMAN. |
| *Ogle County Medical Society,* | Dr. E. M. LIGHT. |
| *Will County Medical Society,* | Dr. A. L. MCARTHUR. |
| *Kane County Medical Society,* (*St. Charles,*) | Dr. W. J. NORTON. |

After calling the roll of permanent members, the Secretary read a letter of resignation from Dr. H. Shoemaker, he having removed from the State.

On motion of Dr. DAVIS, the resignation was accepted.

Dr. DAVIS then offered the following :

*Resolved*, That all regular members of the profession, whether residents of the city or visitors from abroad, be invited to take seats with us, during the meetings of the Society, as members by invitation.

The resolution was adopted.

On motion, it was ordered—

That one member from each County represented, be selected to constitute a Committee for the Nomination of Officers.

The Society took a recess of fifteen minutes, to allow the delegations time to select the Committee, which consisted of the following persons:

| | |
|---|---|
| Drs. JOHN EVANS, | *of Cook County*, |
| C. N. ANDREWS, | *Winnebago County*, |
| W. W. WELCH, | *Lasalle* " |
| A. B. CHAMBERS, | *Peoria* " |
| W. CHAMBERLIN, | *Stark* " |
| S. W. NOBLE, | *Mc Lean.* " |

The Committee on Nominations reported as Officers for the ensuing year for—

PRESIDENT.

DANIEL BRAINARD, *of Chicago.*

VICE-PRESIDENTS.

C. N. ANDREWS, *of Rockford*,

H. NOBLE, *Mc Lean, Co.*

SECRETARIES.

H. A. JOHNSON, *of Chicago*,

W. W. WELCH, *of Lasalle.*

TREASURER.

A. B. CHAMBERS, *of Peoria.*

The report was accepted, and the Committee discharged.

On motion of Dr. BLANEY, the persons named were unanimously elected Officers for the ensuing year.

On motion, a Committee, consisting of Drs. EVANS and McARTHUR, was appointed to conduct the President and Vice-Presidents to their chairs.

The President made a short and appropriate address, in reference to the objects of the Society, and the importance of the interests represented; after which the Society adjourned to meet at 2 o'clock, P. M.

---

*Afternoon Session*, 2 *P. M.*

The Society met, pursuant to adjournment, the President, Dr. BRAINARD, in the Chair.

On motion of Dr. W. O. CHAMBERLIN, it was

*Resolved*, That every motion shall be made in writing, and handed to the Secretary.

On motion of Dr. E. McARTHUR, Dr. Geo. Dennig was made a member by invitation.

The reports of Standing Committees being next in order,

The report of Committee on Surgery was called for; the chairman being absent, no report was presented.

The same was the case in regard to the Committee on Practical Medicine.

Dr. Evans, chairman of the Committee on Obstetrics, being temporarily absent,

On motion of Dr. HERRICK, it was ordered that the reading of the report of this committee be deferred till a future period.

The Committee on Drugs and Medicines being called for, on motion of Dr. HERRICK, it was

*Resolved*, That the Committee have liberty to report hereafter, at such time as the Committee of Arrangement shall designate.

Dr. W. O. CHAMBERLIN moved the following:

*Resolved*, That our next annual meeting be held at Lasalle.

After a discussion, in which Drs. McArthur, Hall, Welch, Blaney, Chamberlin and Cooper participated, it was adopted.

The following gentlemen were proposed and elected permanent members of the Society:

| | | |
|---|---|---|
| Dr. JOSEPH P. ROSS, | *of Chicago*, | proposed by Dr. McARTHUR. |
| S. W. THOMPSON, | *Albion*, | " " |
| A. M. CATLIN, | *Rockford*, | " " |
| W. B. SIMMINGTON, | | " A. L. McARTHUR. |
| R. F. HENRY, | *Princeville*, | " W. CHAMBERLIN |
| CHAS. CUTTER, | " | " " |
| F. D. FITCH, | " | " " |
| E. PINNEY, | " | " " |

| | | | | | | |
|---|---|---|---|---|---|---|
| Dr. | R. McCORMICK, | *of Brimfield,* | *Peoria Co.,* | proposed by | Dr. | G. L. Corcoran. |
| " | DAVID RUTTER, | *Chicago,* | *Cook Co.,* | " | " | Johnson. |
| " | R. B. RUTTER, | " | " | " | " | Herrick. |
| " | P. B. McKAY, | " | " | " | " | Haven, Brainard. |
| " | J. C. BROWN, | *Lasalle,* | *Lasalle Co.* | " | " | Welch. |
| " | H. C. WATSON, | " | " | " | " | |
| " | JOHN GILLETT | " | " | " | " | |
| " | FRANCIS BAY, | " | " | " | " | |
| " | L. D. BOONE, | *Chicago,* | *Cook Co.* | " | " | Herrick and Davis, |
| " | BROCK McVICKAR, | " | " | " | " | |
| " | J. BLOODGOOD, | " | " | " | " | |
| " | JOHN C. MORFIT, | " | " | " | " | |
| " | CHAS. G. SMITH, | " | " | " | " | |
| " | J. W. ELDRIDGE, | " | " | " | " | |
| " | H. PARKER, | " | " | " | " | |
| " | H. R. BELL, | " | " | " | " | |
| " | V. L. HURLBUT, | " | " | " | " | |
| " | MAX MYERS, | " | " | " | " | |
| " | JAS. A. COLLINS, | " | " | " | " | |
| " | R. C. HAMILL, | " | " | " | " | W. E. Clarke. |
| " | WARREN MILLAR, | " | " | " | " | Blaney. |
| " | L. P. CHENEY, | " | " | " | " | McArthur. |
| " | I. P. LYNN, | " | " | " | " | Johnson. |
| " | W. C. HUNT, | " | " | " | " | Davis. Miller. |
| " | C. MARTIN, | *Freeport,* | *Stephenson Co.,* | " | " | L. Clark. |

Dr. Blaney moved the following.

*Resolved,* That all regular members of the medical profession from other States, who may be present in this city during the session, be invited to take seats with the society as members by invitation.

The resolution was adopted.

The appointment of standing committees being next in order, on motion of Dr. Miller it was

*Resolved,* That a committee of one from each county represented at this meeting be appointed by the President to nominate the standing committees for the ensuing year.

The Committee of Arrangements reported Wednesday evening as the time for hearing the annual address, and that the same be delivered in Tremont Hall at 7½ o'clock. They also recommended that the public be invited to attend.

The report was adopted.

Dr. Davis, in behalf of the medical officers of the Illinois Gen.

Hospital, and Dr. HERRICK, in behalf of the U. S. Marine Hospital, extended an invitation to the society to visit those institutions. The invitations were accepted, and to-morrow (Wednesday) morning from 8 to 10 o'clock designated for that purpose.

Dr. HERRICK moved the following:

*Resolved*, That a committee of three be appointed by the Chair, whose duty it shall be to report the unfinished business pending since the last meeting of the society.

The resolution was adopted, and Drs. HERRICK, JOHNSON and COOPER appointed that committee.

Dr. THOS. HALL of the Committee on Prize Essays reported that no communications had been received.

On motion of Dr. E. MCARTHUR it was

*Resolved*, That the report of the Committee on Drugs and Medicines be made the special order of business for to-morrow afternoon at 3 o'clock.

On motion of Dr. HERRICK, it was

*Resolved*, That, when the society adjourn, it adjourn to meet at this place at 10 o'clock to morrow morning.

On motion the society adjourned.

---

### SECOND DAY.

Wednesday, 10 o'clock, A. M., the Society was called to order by the President.

The minutes were read and approved.

The reading and discussion of voluntary communications being in order, the Committee of Arrangements announced a paper on the medical and surgical diseases of the eye by Dr. E. S. COOPER, of Peoria, which was read and discussed by Drs. HALL and BRAINARD.

The Chair appointed the following members, a Committee for Nominating the Standing Committees for the ensuing year:

| | |
|---|---|
| Dr. N. S. DAVIS, | *of Cook County,* |
| W. W. WELCH, | *Lasalle* " |
| A. B. CHAMBERS, | *Peoria* " |
| E. M. LIGHT, | *Ogle* " |
| A. L. MCARTHUR, | *Will* " |
| W. I. NORTON, | *Kane* " |
| LUCIUS CLARK, | *Winnebago County,* |
| WM. CHAMBERLIN, | *Stark* " |
| S. W. NOBLE, | *McLean* " |

Dr. HERRICK presented the following communication:

The undersigned, a Committee in behalf of the Medical Profession of the city of Chicago, invite the members of the profession now visiting our city to an entertainment, to be given at the Tremont House, Thursday evening, at 8 o'clock.

Signed, J. W. ELDRIDGE,

W. B. HERRICK,

B. McVICAR.

D. RUTTER,

MAX MYERS,

} *Committee.*

June 8th, 1853.

On motion of Dr. WELCH, the invitation was accepted.

On motion of Dr. W. CHAMBERLIN, the Society adjourned to meet at 2½ o'clock. P. M.

---

*Afternoon Session*, 2½ *P. M.*

The Society was called to order by the President.

Dr. BLANEY, chairman of the Committee on Drugs and Medicines, read a report, and, in conclusion, offered the following resolutions:

1. *Resolved*, That the Illinois State Medical Society have learned with much pleasure of the Institution of the National Pharmaceutical Association, and would hereby express a desire and intention, as far as may be, to co-operate with the laudable exertions of that body, in the advancement of pharmaceutical knowledge, and elevation of the professional character of apothecaries and druggists throughout the United States.

2. *Resolved*, That this Society recommend to druggists and apothecaries in Illinois, that, as far as circumstances will admit, they form among themselves pharmaceutical societies, to enable them to act in the most efficient manner as adjuvants to the National Pharmaceutical Association, in the work of elevation and reform of their profession.

3. *Resolved*, That we, the members of the Illinois State Medical Society, will, individually and collectively, exert our utmost influence to procure the more thorough education of apothecaries and druggists in this State; and, as far as practicable, trade only with those whose honesty and knowledge of their business *deserve* our patronage.

4. *Resolved*, That we pledge ourselves not to purchase drugs or medicinal preparations, without rigid scrutiny as to their quality and freedom from sophistication, and invariably refuse to purchase any but the purest and best articles we can obtain.

5. *Resolved*, That the District, County, and City Societies be recommended to discuss the expediency of adopting the third and fourth resolutions appended to this report.

6. *Resolved*, That the District, County and City Societies be recommended each to appoint one or more members of their respective bodies, examiners of drugs and medicines offered for sale in their respective cities, or districts, and that they require from said examiners a semi-annual report of the condition of the drug market in their respective districts; and that said examiners shall, as early as April 1st, of each year, furnish to the chairman of the Committee of Drugs and Medicines of the Illinois State Medical Society, all important facts which may come to their knowledge in relation to the sophistication and sale of drugs and medicines in the State of Illinois.

7. *Resolved*, That this Society cordially adopt the following two resolutions of the National Pharmaceutical Convention, held at Philadelphia, October 6th, 1852, viz: "*Resolved*, That in the opinion of this Convention, the law against the importation of adulterated drugs, chemicals, and medicinal preparations, has already effected much good, by excluding large quantities of inferior drugs from the market. *Resolved*, That, inasmuch as the usefulness of the law will be proportioned to the ability, and conscientious discharge of duty, of examiners, that this Convention shall respectfully and urgently represent to the appointing power the cardinal importance of preventing the removal of qualified examiners on mere political grounds."

8. *Resolved*, That a committtee of —— be appointed by the Chair to correspond with the National Pharmaceutical Association, and co-operate with that body in carrying into effect the spirit of the last quoted resolution.

All of which is respectfully submitted.

JAMES V. Z. BLANEY, Chairman.

On motion of Dr. PADDOCK, the report was adopted and referred to the Committee of Publication, reserving the resolutions for discussion.

The resolutions were then taken up seriatim, and adopted.

Dr. PADDOCK moved that the blank in the eighth resolution be filled by one.—Carried.

Dr. Blaney was appointed by the Chair, the Committee designated in the last resolution.

On motion of Dr. LYMAN, it was

*Resolved*, That the chairman of the Committee on Drugs and Medicines be requested to give verbally, as an addition to his report any facts or principles he may be in possession of, relating to anesthesia, and the use of chloroform and ether.

On motion of Dr. W. E. Clarke, it was

*Resolved*, That Dr. Blaney's communication be made the first order of business to-morrow (Thursday) afternoon.

Dr. McArthur read a communication from Dr. Saml. Thompson, of Albion, on the use of Turpentine.

On motion of Dr. Arnold, it was

*Resolved*, That this valuable communication be received, and referred to the Committee of Publication; and that the thanks of the Society be tendered to Dr. Thompson.

Dr. Thompson's communication also called the attention of the Society to the subject of legislation in regard to the sale of medical compounds and nostrums, and also suggested that this society take some action concerning the conditional granting of degrees by medical colleges.

On motion of Dr. McArthur, it was

*Resolved*, That a committee of three be appointed by the Chair to take into consideration the two subjects alluded to by Dr. Thompson, and report to the Society to-morrow.

The following gentlemen were appointed that committee:

Dr. E. McArthur, Dr. J. D. Arnold,
W. Lyman.

On motion of Dr. Norton, it was

*Resolved*, That the thanks of the Society be tendered to Prof. Davis, and the Trustees of the Illinois General Hospital; and to Prof. Herrick, and the officers of the U. S. Marine Hospital, for thoir attentions during the visits of the Society to those institutions this morning.

On motion of Dr. McArthur, the Society then adjourned until to-morrow morning, at 9 o'clock.

---

Wednesday evening, the annual address before the Society, was delivered by Prof. N. S. Davis, in Tremont Hall.

The subject, "The Intimate Relation of Medical Science to the whole field of Natural Sciences."

---

### THIRD DAY.

*9 o'Clock, A. M.*

The Society met, pursuant to adjournment, Vice-President Dr. Noble, in the Chair.

Minutes of yesterday's proceedings read and approved.

Dr. Joseph Stout, of Ottawa, was proposed by Dr. Welch, and elected a permanent member of the Society.

On motion of Dr. Andrews, it was

*Resolved*, That Dr. A. L. McArthur, of Joliet, be appointed to deliver the annual public address before the society, at its next regular meeting.

Dr. Welch offered the following:

*Resolved*, That each local society, and the profession generally, throughout the State, be recommended to send petitions to the next legislature, for the passage of laws regulating the practice of medicine.

After some discussion, on motion of Dr. W. Chamberlin, the resolution was laid on the table.

The Committee, to whom were referred the suggestions of Dr. Thompson, in reference to procuring the action of our legislature, in regard to the sale of medical compounds and nostrums; and also in regard to the action of this Society concerning the conditional granting of degrees by medical colleges; although sympathizing with Dr. Thompson in the object desired, nevertheless believe that it is inexpedient for this Society at the present time to take any action on the subject.

In behalf of the Committee,

E. McARTHUR, *Chairman.*

On motion, the report was accepted and the committee discharged.

On motion of Dr. Thos. Hall, it was

*Resolved*, That the Secretary of each county or local medical society be appointed a Committee of one to report on the most prevalent diseases of his county or district during each medical year, and that he be earnestly requested to forward the same to the Chairman of the Committee on Practical Medicine of this Society, at least one month before the annual meeting.

The Treasurer's report was read by Dr. Thomas Hall, and, on motion of Dr. E. McArthur, it was

*Resolved*, That a committee of three be appointed by the Chair to examine the vouchers and report this afternoon; also

That the same Committee report the annual assessment necessary for defraying the expenses of the current year.

Drs. E. McArthur, Arnold and Hall were appointed that committee.

Dr. Davis, from the Committee for Nominating the Standing

Committees, made the following report; which, on motion of Dr. LYMAN, was accepted and adopted.

| | |
|---|---|
| *Committee of Arrangements,* | Drs. W. W. WELCH, of Lasalle,<br>J. C. BROWN, "<br>H. C. WATSON, " |
| *Committee on Surgery,* | Drs. W. B. HERRICK, of Chicago,<br>J. D. ARNOLD, of Peoria,<br>F. A. McNEILL, of Mt. Morris. |
| *Committee on Practical Medicine,* | Drs. LUCIUS CLARK, of Rockford,<br>A. M. CATLIN, "<br>S. W. NOBLE, of Leroy. |
| *Committee on Obstetrics,* | Drs. THOMAS HALL, of Toulon,<br>W. CHAMBERLIN, "<br>A. B. CHAMBERS, of Peoria. |
| *Committee on Drugs & Medicines,* | Drs. DE LASKIE MILLER, Chicago,<br>A. L. McARTHUR, of Joliet,<br>SAML. THOMPSON, of Albion. |

Dr. ANDREWS, read a paper on the Development of the Placenta in the Fallopian tubes, with reports of cases.

On motion of Dr. HALL, it was

*Resolved*, That the thanks of the Society be tendered to Dr. Andrews for his interesting paper; and that the same be referred to the Committee of Publication.

Dr. BRAINARD, in compliance with the request of the Committee of arrangements, read from his case book notes of two cases of obliteration of the vagina; also a case of cartilaginous exostosis of the condyle, ramus and angle of the lower jaw, removed by operation. The morbid specimen was presented for inspection.

Dr. PADDOCK introduced the following, which was adopted:

*Resolved*, That the thanks of the Society be tendered to Dr. Brainard for his reports, and that he be requested to place them at the disposal of the Committee of Publication.

Dr. EVANS presented the following

*Resolved*, That the constitution be so amended as to require the Standing Committee on Obstetrics to report upon the improvements and important suggestions in the science and art of obstetrics, and the pathology and treatment of diseases of women and children, that have been made within the state of Illinois during the year, or not previously noticed in the proceedings of this Society.

On motion of Dr. E. MCARTHUR, the Society adjourned to meet at 2½ o'clock, P. M.

---

*Afternoon Session, 2½ P. M.*

The Society met, and was called to order by the President.

The Committee appointed to examine the report of the Treasurer reported that they had found the same correct, and they recommended an assessment of two dollars on each member, for the purpose of defraying the current expenses of the Society.

The report was accepted and adopted.

In accordance with the request of the Society, Dr. BLANEY pro ceeded to give his verbal report on the use of anesthetics.

In the discussion of the subject, Drs. Brainard, Blaney, W. O. Chamberlin and Hall participated.

Dr. W. O CHAMBERLIN presented the following, which was adopted :

*Resolved*, That the thanks of the Society be presented to Dr. Blaney, for his able report, and that he be requested to embody his remarks this afternoon, in connection with his previous report, for publication.

On motion of Dr. THOS. HALL, it was

*Resolved*, That the subject proposed at the last annual meeting for prize essays be continued another year, and that a committee be now appointed to receive essays and award the premium.

The Committee of last year was continued, substituting Dr. Arnold for Dr. Rouse.

The following members of the society were elected delegates to represent this Society at the next annual meeting of the American Medical Association, to meet in St. Louis, in May next.

Dr. SAMUEL THOMPSON, of Albion,
" H. A. JOHNSON, of Chicago,
" THOS. HALL, of Toulon,
" LUCIUS CLARK, of Rockford.
" NATHANIEL ENGLISH, of Jacksonville,
" A. L. McARTHUR, of Joliet,
" SAMUEL LONG, of Springfield,
" DeLASKIE MILLER, of Chicago,
" EDWIN DICKINSON, of Peoria,
" S. A. PADDOCK, of Princeton,
" J. S. WHITMIRE, of Metamora,
" W. W. WELCH, of Lasalle.

On motion of Dr. EVANS, the delegates were empowered to appoint substitutes in case of their own inability to attend.

Dr. HALL read an interesting paper on mucous diarrhœa and puerperal fever; he also made some remarks on citric acid in rheumatism, sulph. acid in cholera, with details of other cases.

On motion of Dr. WELCH, it was

*Resolved*, That the thanks of the Society be tendered to Dr. HALL, and that his communication be referred to the Committee of Publication.

On motion of Dr. EAVNS, it was

*Resolved*, That the thanks of the Illinois State Medical Society be tendered to the Common Council of the City of Chicago for the use of their council chamber for the sittings of the society during its present session.

On motion of Dr. ANDREWS, it was

*Resolved*, That the thanks of the Illinois State Medical Society be tendered to the Medical Profession of the City of Chicago for the ample accommodations furnished for the society, and for the kind and liberal hospitality extended towards its members during its present session.

On motion of Dr. L. A. MCARTHUR, the thanks of the society were tendered to Dr. DAVIS for his interesting address, and a copy requested for publication in the transactions.

On motion of Dr. HERRICK, the society adjourned.

